# The Efficacy of Intradermal Injection of Botulinum Toxin in Patients with Post-Herpetic Neuralgia

**Published:** 2011-05-01

**Authors:** M R Emad, M Emad, P Taheri

**Affiliations:** 1 Department of Physical Medicine and Rehabilitation,Faghihi Hospital, Shiraz University of Medical Sciences, Shiraz, Iran; 2 Department of Dermatology, Faghihi Hospital, Shiraz University of Medical Sciences, Shiraz, Iran

**Keywords:** Post herpetic neuralgia, Pain, Botulinum toxin

## Abstract

**Background:**

Several treatments have been suggested in shingles viral infection caused by Varicella zoster virus that may lead to complications such as PHN (Post-herpetic neuralgia). Intradermal injection of botulinum toxin was shown with few side effects. This study evaluates the efficacy of intradermal injection of botulinum toxin in patients suffering from PHN.

**Methods:**

Fifteen patients suffering from PHN for more than1 month were enrolled. Data collected were patients' age, sex, and lesion site, the dermatome involved and the duration and severity of pain by visual analog scale (VAS). Botulinum (15 units /every 10 cm(2) of body involved) was injected intradermally. The patients were followed 2, 14 and 30 days after injection.

**Results:**

Of participants, 6 were males and 9 females. The mean age was 60 years and the mean duration of neuralgia was 6.5 months. The mean VAS on day 2 was 6.4, on day 14 was 7.2 and after 30 days was 7.6. The overall pain after injection decreased but was not significant.

**Conclusion:**

It seems that intradermal injection of botulinum toxin decreases pain in PHN patients and this decrease is less prominent by passing time.

## Introduction

Zona occurs due to weakening of immune system, increase in age, morbidity, chemotherapy, etc. Its prevalence in normal population less than 20 years is 1/1000 and is 5-10 times higher in those above 80 years of age.[1] Post-herpetic neuralgia (PHN) is the most common complication of zona which is more commonly seen in the elderly population than the children. The frequency of zona in the population under 60 years of age is 2%.[2] In a study by Oxman et al., the prevalence of zona between the age group 60- 69 years was reported to be 6.9% and in the population above 70 years of age it was 18.5%.[3]

PHN is divided into three phases of acute (first month after the onset of rash), sub-acute (one to four months after the onset) and chronic (> 4 months after the onset)[4] and mostly involves the trigeminal, cranial and thoracic nerves.[5] The pain caused by PHN is either continuous with a burning sensation or alternating with stinging sensation.[6] Almost, 90% of the people suffering from PHN show an unusual response to normal painless stimuli, i.e. they develop pain upon exposure to light and touch.[7] The pain and discomfort due to PHN can be so severe that sometimes it can lead to sleep disturbances, decreased appetite, and decreased libido.[5] The pain can be so severe that might lead to suicide.

One of the reasons for the constant pain months after zona infection is the increase in the number of P fibers and decrease in the number of the large nerve fibers which suppress the pain transmission.[8] One of the treatments recommended for PHN is the use of botulinum toxin type A. Few studies performed in this regard have shown that intradermal injections have analgesic effects on PHN patients. The problem arising during the treatment of PHN is that the medication prescribed should have few or no side effects and should not cause cognitive impairment in the patients[9][10] and most of patients suffering from PHN are elderly and have other comorbidities. Treatment of PHN with botulinum toxin type A has shown promise in the long term pain control without any cognitive side effects.[11] In this study, we aimed to evaluate the effects of botulinum toxin type A in PHN patients.

## Materials and Methods

In this interventional study, 15 patients suffering from PHN were selected using simple sampling method. The patients were included if they were over 18 years of age, had developed PHN at least one month after the rash, had not used botulinum toxin type A previously, had no coagulopathies and localized infection, no history of allergy to analgesics, did not have any tumor at the site, did not have septicemia, were not pregnant or lactating, did not have psychiatric problems or mental retardation, did not use amino glycosides, and did not suffer from myasthenia gravis.

After selection of patients, required explanations regarding the features of the drug, method of injection, possible complications of injection, possible analgesic effects were provided for patients. A consent form was taken from each patient. A questionnaire was used including data on age, sex, address, chief complaint, site of lesions (rash), the dermatome involved, duration of PHN and severity of symptoms determined through a Visual Analogue Scale (VAS) as a standard criteria for determining the severity of pain.[12] The selected cases had a past medical history of affliction with zona, had not responded to the routine treatments for disease, and had referred to the psychiatry and dermatology clinics of Shiraz University of Medical Sciences.

Before starting the intradermal injection of botulinum toxin type A, 2% lidocaine gel was applied on the site. After applying the gel, botulinum toxin A specific for every patient was injected based on our selected pattern. Fifteen units of botulinum toxin A diluted with 2% lidocaine were injected intradermally per 10 cm(2) of the painful surface area using the syringes used for injecting insulin. For patients, botulinum toxin type A with the trade name of dysport was used and each vial of the toxin was diluted with 4 ml of 2% lidocaine. The amount of toxin was different for every patient depending upon the extent and site of the lesion. The patients were followed after 2, 14 and 30 days and the leftover pain was measured using VAS. Injections of the toxin and the follow-ups were done by one person. All data were statistically analyzed using SPSS software (Version15, Chicago, IL, USA). In order to measure the effects of the toxin, Repeated Measure ANOVA was used and 0.05 was considered as the significance level.

## Results

Out of 15 patients, 6 were male and 9 were female. The minimum age was 50 years and the maximum 67 years with the mean of 60 years. The minimum duration of PHN was 1 month and the maximum was 36 months with the mean value and standard deviation of 6.4±8.4. The dermatomes involved ranged from 2-7 with a mean of 4 dermatomes. VAS was measured during the follow-up visits of the patients. On day 2, VAS was between 0-10 with the standard deviation of 6.4±4.4. In the second week, it was measured to be between 0-10 with the standard deviation of 4.2±7.2. Similarly, VAS was measured in the fourth week with minimum and maximum values of 0-10 and standard deviation of 3.7±7.6.

**Fig. 1:**
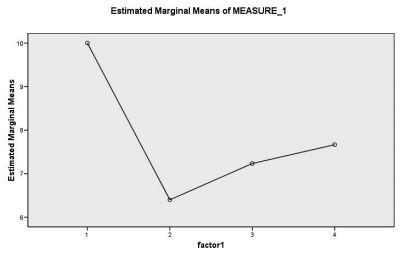
Changes based on VAS, pain before the injection, the second day, second week and first month after the injection of botulinum toxin A

Figure 1 shows the changes in VAS after the injection of botulinum toxin on days 2, 14 and 30 after treatment. Pain decreased in patients in the fourth week in comparison to the pre-treatment period which was not statistically significant (p=0.06). Pain on the second day (p=0.007), in the second week (p=0.02) and in the first month (p=0.03) showed a significant decline as compared to pre-treatment group. The second week, a slight increase was noticed in pain as compared to the second day (p=0.16). In the fourth week, an increase in pain was also observed when compared to the second day (p=0.06). Also, increase in pain in the fourth week as compared to the second week was not significant (p=0.13).

## Discussion

The pain caused by PHN has a very complex mechanism, so different treatments have been suggested including routine analgesics, tricyclic antidepressants, carbamazepine, gabapantine, perfenazine, haloperidol, phenophenazine, aspirin, narcotics, cognitive techniques like relaxation, hypnotism and bio feedbacks, and invasive techniques like surgery (blocking the nerve supplying that dermatome or stimulating the posterior spinal cord).[13][14][15] Using botulinum toxin for treatment of neuralgias is a recent technique which has been used for the treatment of migraine headaches,[16][17][18][19][20] tension headaches,[21][22][23][24][25] back aches, and myofacial pain.[26][27] This combination of treatments can be useful in neuropathic and nociceptive pain suppression.[28]

After reactivation, zona causes inflammation of the posterior ganglionic roots along with changes in the nociceptive pathways with automatic discharges and decrease in the nerve transmission which leads to the neuropathic pain. On the other hand, the nervous system becomes very sensitive to pain and develops pain even when in contact with weak stimuli.[6][29][30][31] Allodynia pain may be due to neural contacts which transmit pain to the central nervous system. Hyperactivity

of the efferent fibers A-beta after the neuronal damage causes pain upon touch, and presynaptic afferent fibers C causes automatic pain. The mechanism of allodynia is not clearly known yet.[32] Intradermal injection of botulinum to decrease burning central pain and allodynia involves the spinal cord. This drug decreases the neuropathic pain by affecting the periphery and most probably decreasing the central sensitivity.[33] The analgesic pharmacological effect of intradermal injection is due to the release of neurotransmitters like acetylcholine from the axon terminals of the motor neurons, parasympathetic neurons, presynaptic sympathetic neurons and post-ganglionic parasympathetic neurons with different mechanisms.[34]

Intradermal injection of botulinum decreases the substances with nociceptive sensitivity. Studies have shown that nociceptive effect of the localized injection of botulinum toxin leads to the inhibition of the release of formalin-induced glutamate, P substance and calcitonin gene-related peptide which play an important role in the inflammation of neurons.[35][28] On the whole, neuropathic analgesic effect of botulinum toxin on PHN is due to the direct effect on sensory neurons and indirect effect on central nervous system.[36] This study showed that intradermal injection of botulinum toxin decreases pain in PHN but its analgesic effect lessens with the passage of time e.g. those who had received botulinium toxin, 2 weeks or one month before had experienced more pain as compared to those who received the injection just 2 days ago. One month after the injection, the patients experienced less pain and had better quality of life in comparison. Freund and Schwarts in a study done on 7 patients who suffered from PHN showed that botulinum injections decreased pain too. These patients had pain in the trigeminal, thoracic and back regions. Before the injection, their mean VAS was 8 which decreased to 5 after treatment. The patients who suffered from trigeminal neuralgia had responded much better to the treatment.[37]

A study was done on an 80 year old male suffering from PHN, who had co-morbidities like coronary heart disease and heart failure. The oral medications given to the patient were not effective as they caused delirium and cognitive dysfunction. Intradermal botulinum injections were given which were very effective as they decreased the pain of the patient from VAS=10 to VAS=1 and the analgesic effect of the injection lasted for 52 days.[38]

Another study done on 3 patients suffering from PHN showed that botulinum injections decreased VAS from 8 to 2 in the second week of the follow up. In the tenth week, pain increased as compared to the second week but it was bearable.[39] Similarly, Rusi Huete performed a study on an 83 year old female suffering from PHN. She had received botulinium toxin injection and showed a very good response to the treatment without any side effects.[40]

Considering the present and some previous studies done on the effect of botulinum toxin in patients suffering from PHN, it seems that there can be a decrease in pain in the first week. However, analgesic effects of botulinum toxin decrease gradually as the weeks pass by. Overall, the pain is less and more tolerable when compared to pre-treatment period. This problem was reported in all studies. Due to the low number of available eligible patients, control group was not included in this study and it is suggested that a random clinical trial should be performed in which a group of patients receive placebo so that the analgesic effects of botulinum in the treatment of PHN can be better evaluated.
